# Dual Anti-Malarial and GSK3β-Mediated Cytokine-Modulating Activities of Quercetin Are Requisite of Its Potential as a Plant-Derived Therapeutic in Malaria

**DOI:** 10.3390/ph14030248

**Published:** 2021-03-09

**Authors:** Amatul Hamizah Ali, Suhaini Sudi, Ng Shi-Jing, Wan Rozianoor Mohd Hassan, Rusliza Basir, Hani Kartini Agustar, Noor Embi, Hasidah Mohd Sidek, Jalifah Latip

**Affiliations:** 1 Department of Chemical Sciences, Faculty of Science and Technology, Universiti Kebangsaan Malaysia, Bangi 43600, Selangor, Malaysia; amatulhamizahali@yahoo.com.my; 2 Department of Biological Sciences and Biotechnology, Faculty of Science and Technology, Universiti Kebangsaan Malaysia, Bangi 43600, Selangor, Malaysia; suhainisudi@gmail.com (S.S.); shijing9147@hotmail.com (N.S.-J.); noormb@ukm.edu.my (N.E.); hasidahms@gmail.com (H.M.S.); 3 Department of Biomedical Sciences and Therapeutics, Faculty of Medicine and Health Sciences, Universiti Malaysia Sabah, Kota Kinabalu 88400, Sabah, Malaysia; 4 School of Biological Sciences, Faculty of Applied Sciences, Universiti Teknologi MARA, Shah Alam 40450, Selangor, Malaysia; rozianoor@uitm.edu.my; 5 Pharmacology Unit, Department of Human Anatomy, Faculty of Medicine and Health Sciences, Universiti Putra Malaysia, Serdang 43400, Selangor, Malaysia; rusliza@upm.edu.my; 6 Department of Earth Sciences and Environment, Faculty of Science and Technology, Universiti Kebangsaan Malaysia, Bangi 43600, Selangor, Malaysia; hani_ag@ukm.edu.my

**Keywords:** cytokine, GSK3β, malaria, *Plasmodium berghei*, quercetin

## Abstract

Although death in malaria is attributed to cerebrovascular blockage and anaemia, overwhelming cytokine production can contribute to the severity of the disease. Therefore, mitigation of dysregulated inflammatory signalling may provide further benefit for malaria treatment. Quercetin (3,3′,4′,5,7-pentahydroxyflavone) is known to inhibit glycogen synthase kinase-3β (GSK3β), a potent regulator of both pro- and anti-inflammatory effects. Quercetin is therefore a potential therapeutic to modulate the imbalanced cytokine production during malarial infection. Anti-malarial effects of quercetin were evaluated in murine models of severe and cerebral malaria using *Plasmodium berghei* NK65 and ANKA strains, respectively. Western blotting and analysis of cytokines were carried out to determine the GSK3β-mediated cytokine-modulating effects of quercetin in infected animals. Quercetin (25 mg/kg BW) treatment in *P. berghei* NK65-infected animals resulted in 60.7 ± 2.4% suppression of parasitaemia and significantly decreased serum levels of TNF-α and IFN-γ, whilst levels of IL-10 and IL-4 were elevated significantly. Western analysis revealed that pGSK3β (Ser9) increased 2.7-fold in the liver of quercetin-treated NK65-infected animals. Treatment of *P. berghei* ANKA-infected mice with quercetin (15 mg/kg BW) increased (2.3-fold) pGSK3β (Ser9) in the brains of infected animals. Quercetin is a potential plant-derived therapeutic for malaria on the basis that it can elicit anti-malarial and GSK3β-mediated cytokine-modulating effects.

## 1. Introduction

Aberrant cytokine production, or a “cytokine storm”, can cause severe systemic inflammatory responses as well as tissue and organ damage in various viral, bacterial, and parasitic infections [1]. Drug-discovery efforts are now not only focusing on eliminating the causative agents of the disease but also on immunomodulatory activities in the host to mitigate the overwhelming cytokine production [2]. In the case of malaria, uncontrolled and excessive immune responses mounted by the host in an attempt to eliminate the parasite contribute significantly to the pathology of the disease [3]. Inflammatory cytokines such as TNF-α have important roles in the pathophysiology of cerebral malaria based on murine models of infection. For example, increased systemic TNF-α cytokine level and sequestration of parasitised erythrocytes in the brain can lead to coma and consequent death [4]. Excessive cytokines have also been associated with susceptibility to severe malarial anaemia by perturbing erythropoiesis. TNF-α inhibits major erythroid transcription factor GATA-1 expression and further inhibits erythroblast differentiation [5]. Modulation of immune-mediated inflammatory responses is, therefore, also significant in the therapeutic approach against malarial inflammation. Host signalling kinases activated by malarial infection represent attractive targets for anti-malarial intervention [6]. Many therapeutic effects of plant extracts and their bioactive compounds are also attributed to their immunomodulatory effects and influence on the host immune system [7]. Immunomodulation using plant-derived products is a plausible effective strategy in the development of adjunctive therapy, such as for the mitigation of the cytokine storm during pathogenic infections. Flavonoid constituents in various medicinal plants are known to possess a wide range of pharmacological activities, including anti-inflammatory effects. For example, the flavonol quercetin (3,3′,4′,5,7-pentahydroxyflavone), an anti-oxidant and a common naturally-occurring phytochemical, exerts a marked effect on inflammation [8,9,10,11].

Quercetin inhibits the activation of nuclear factor-κB (NF-κB), a transcription factor that regulates multiple aspects of innate and adaptive immune functions and thus serves as a pivotal mediator of inflammatory responses [12,13]. Quercetin is reported to significantly down-regulate TNF-α, IL-1β, and IL-6 and attenuate the inflammatory response in LPS-induced RAW264.7 cells [14]. The current coronavirus (COVID-19) pandemic has created much attention and immense effort worldwide in the search for drugs (e.g., immunomodulators) that can reduce viral load and control inflammatory responses [15,16]. In this respect, Rudd [17] hypothesises that GSK3β inhibition is a plausible therapeutic approach against COVID-19, capitalising on the dual benefits of inhibiting viral replication while potentiating the immune response. For example, quercetin as an anti-viral agent [18,19] is also known to exhibit in silico and in vitro inhibition of glycogen synthase kinase-3β (GSK3β) [20,21]. Subsequently, Jung et al. [22] reported that flavonoids, including luteolin, apigenin, and quercetin, inhibit GSK3β. Recently Friday et al. [23] also reported that quercetin and its analogues are inhibitors of GSK3β based on a study using pharmacophore modelling and molecular-docking techniques. This Ser/Thr kinase is a molecular hub linking numerous signalling pathways in the cell, including host-directed inflammatory responses. Thus, quercetin with its anti-inflammatory effects is a plausible therapeutic candidate for mitigating the cytokine storm [24]. It is noteworthy that the anti-parasitic activity of several naturally derived products has been attributed to the presence of quercetin or derived compounds [25]. There have been various reports on the anti-plasmodial activity of quercetin [26] and its analogues against clinical isolates of *Plasmodium falciparum* [27]. Recently, it was demonstrated that selected existing commercial flavonoids (including quercetin)-containing drugs for other diseases are active in malaria-infected mice and in vitro against chloroquine-resistant *P. falciparum* [28]. It was suggested that the anti-oxidant capacity of the tested drugs could be responsible for the reduction of malaria severity. Treatment by combining flavonoids, known for their anti-oxidant and anti-inflammatory properties, with standard anti-malarial drugs protected mice infected with *P. berghei* [29]. Taken together, quercetin as an inhibitor of GSK3β may be useful to modulate immune responses during malarial infection.

Glycogen synthase kinase-3 (GSK3), initially described by Embi et al. [30] as a critical enzyme capable of phosphorylating, and consequently inhibiting glycogen synthase during insulin signalling, is now recognised as a point of convergence for host inflammatory response [31,32]. The pathophysiology of many diseases, including bipolar disorder, diabetes mellitus, Alzheimer’s disease, inflammation, and cancer, involves dysregulation of this kinase [33]. In mammals, GSK3 is encoded by two highly related genes encoding GSK3α and GSK3β isoforms of the enzyme, respectively. GSK3 is constitutively active under basal conditions; its regulation is attained mainly via the inhibitory serine-phosphorylation at Ser21 for GSK3α, and Ser9 for GSK3β [34]. Malfunction of GSK3β is implicated in the pathogenesis of several diseases [35]. GSK3β is also associated with the innate-immune response against pathogens [32], and thus GSK3β is a potential target for therapeutic intervention. During infections, GSK3β regulates the production of several pro-inflammatory cytokines while suppressing the production of anti-inflammatory cytokines [32]. Excessive production of pro-inflammatory cytokines during malarial infection can lead to liver and kidney injuries and consequent death [36,37].

Bioactive compounds with dual anti-malarial and anti-inflammatory properties not only may be beneficial in eliminating the plasmodial parasite, but also in modulating the cytokine response during malarial infection [2]. Based on the current understanding described above on the anti-inflammatory and GSK3β-inhibitory activities of quercetin, we propose to evaluate whether the anti-malarial activity of quercetin in a mouse model of malarial infection also involves modulation of inflammatory cytokine response. Findings from the present study may give further insight into whether this flavonol is a plausible candidate in the development of adjunctive therapeutics to mitigate the cytokine storm during malarial infection.

## 2. Results

### 2.1. Quercetin Exhibits Moderate Anti-Plasmodial Activity

In vitro anti-plasmodial assessment of quercetin showed an IC_50_ value of 19.31 ± 1.26 μM against the chloroquine-sensitive strain of P. falciparum 3D7. This value is considered as moderate anti-plasmodial activity based on classification by Dolabela et al. [38]. In comparison, chloroquine (CQ), the reference anti-malarial drug showed potent anti-plasmodial effect (IC_50_ = 0.0023 ± 0.0001 μM). For cytotoxic evaluation, quercetin showed minimal toxicity towards mammalian Chang liver cells (IC_50_ = 868.22 ± 3.81 μM). A selectivity index (SI) value exceeding 10 for quercetin (SI = 44.9) suggests that this compound is selective for the parasite and thus a plausible anti-plasmodial compound.

### 2.2. Quercetin Displayed No Adverse Effect in Non-Infected Mice

All non-infected mice showed 100% survivability until day 30 after being injected (i.p.) with 2.5, 5, 10, 15, 25, and 50 mg/kg BW quercetin. During the 30-day observation period, most non-infected animals did not show any adverse behavioural and physical changes as compared to the control group. Mice from the 50 mg/kg BW group however displayed mild toxicity (lethargy and reduction in locomotor activities). This finding indicates that doses of quercetin of up to 25 mg/kg BW tested here may be further employed for the subsequent four-day suppressive test.

### 2.3. Quercetin Suppressed Parasitaemia Development and Prolonged Median Survival Time in P. berghei NK65- and ANKA-Infected Animals

**Figure 1:**
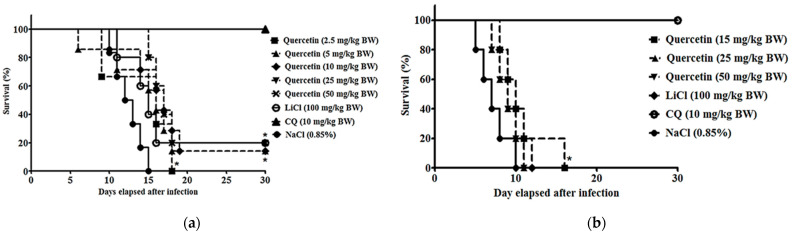
Representative Kaplan–Meier survival curve during the four-day suppressive test in (**a**) *P. berghei* NK65-infected mice and (**b**) ANKA-infected mice with and without quercetin. Figures show survival data for NaCl-treated (negative control) (*n* = 7), quercetin-treated (n = 7), CQ-treated (*n* = 7), and LiCl-treated (*n* = 7) mice. Significant differences between tested and control groups were determined at *p*< 0.05 (*).

*P. berghei* NK65-infected mice treated with quercetin at 25 mg/kg BW displayed the highest chemo-suppressive activities, 60.7 ± 2.4% (*p* < 0.05) (Table 1; Figure 1). Quercetin was able to exhibit dose-dependent anti-malarial activity from 2.5 to 25 mg/kg BW. Suppressive activity was slightly reduced at 50 mg/kg BW. The reduction of activity might be due to quercetin-caused mild toxicity at 50 mg/kg BW. Detoxification mechanisms in experimental animals to excrete the drug from the animals may result in reduction of the drug activity [39]. However, median survival time for doses of 25 and 50 mg/kg BW were the highest (17 days) as compared to controls without treatment (13 days). For *P. berghei* ANKA infection, treatment with quercetin (15 mg/kg BW) in mice resulted in the highest chemo-suppressive effects of 36.10% (*p* < 0.05) compared to the non-treated infected group (Table 1; Figure 1). Chemo-suppressive effects from all doses used in the study that did not exceed 50% may be due to the pathogenicity and severity of the ANKA strain commonly known as a cerebral parasite, which usually targets the brains of the infected animals at the early stage of the infection [40,41]. However, improvement of the median survival time can be significantly observed in mice treated with quercetin 15 mg/kg BW (10 days) as compared to controls without treatment (7 days). The CQ-treated group for NK65 and ANKA infections exhibited the highest chemo-suppressive activities (96.0% and 94.3% respectively) compared to other treatment groups, consistent with its function as an established anti-malarial reference drug. As for LiCl (GSK3 inhibitor reference compound), chemo-suppressive values were significant for both NK65 and ANKA infections (69.4% and 85.2%, respectively).

### 2.4. Quercetin Resulted in Increased GSK3β (Ser9) Phosphorylation in the Liver of P. berghei NK65-Infected Mice

**Figure 2:**
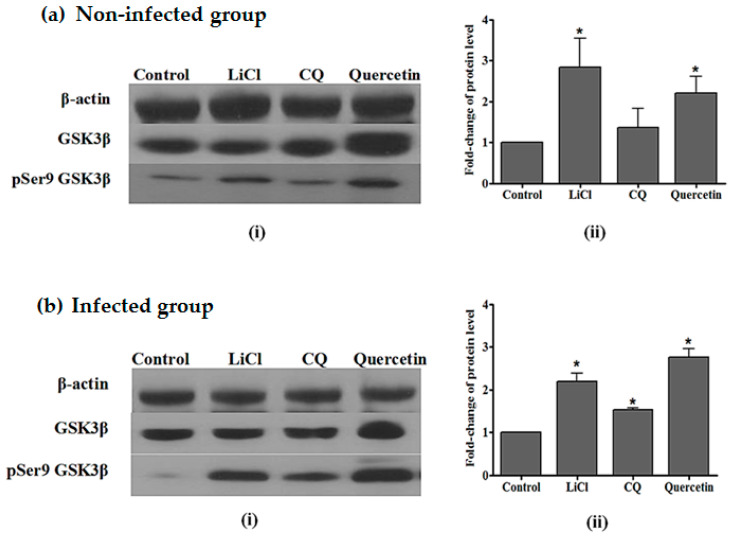
(**a**) (i) Intensity of immunoreactive bands for pSer9 GSK3β and GSK3β (ii) Fold change of phosphorylated protein levels in liver of non-infected mice; (**b**) (i) Intensity of immunoreactive bands for pSer9 GSK3β and GSK3β (ii) Fold change of phosphorylated protein levels in liver of mice infected with *P. berghei* NK65 treated with LiCl, CQ, or quercetin for four days. Total GSK3β protein expression levels were normalised to β-actin, and pGSK3β was normalised to total GSK3β protein level. Densitometric measurements are illustrated as mean (fold) ± SD of corresponding measurements in non-treated controls. Significant differences between test and control groups were measured at *p* < 0.05 (*).

Western-blot analysis from non-infected groups showed the presence of GSK3β and pSer9 GSK3β in liver samples, as seen from the immunoreactive-GSK3 and -pSer9 GSK3 protein bands. The intensities of the immunoreactive bands of GSK3β were uniform, indicating that the total GSK3β protein levels were the same in treated and control groups. Animals treated with quercetin and LiCl each showed significant increases in pSer9 GSK3β with an increment of 2.3- and 2.7-fold, respectively, compared to the control group. The intensities of immunoreactive bands for pSer9 GSK3β were higher for quercetin- and LiCl-treated groups as compared to control groups. Meanwhile, immunoreactive-pSer9 GSK3β protein bands for CQ showed low intensities as compared to quercetin- and LiCl-treated groups (Figure 2).

As for *P. berghei* NK65-infected groups, western analysis of liver samples showed that GSK3β and pSer9 GSK3β were present as immunoreactive-GSK3β and -pSer9 GSK3β protein bands were detected. Uniform intensities in immunoreactive-GSK3β protein bands indicate the same level of total GSK3β protein in both treated and control groups. Figure 2 shows that there was a significant fold-change increase of pSer9 GSK3β protein in the liver samples of infected mice. *P. berghei* NK65-infected mice treated with quercetin and LiCl each showed significant increases in the fold change of pSer9 GSK3β (2.7- and 2.2-fold increment, respectively, compared to non-treated controls).

### 2.5. Quercetin Resulted in Increased GSK3β (Ser9) Phosphorylation in Brains of P. berghei ANKA-Infected Mice

**Figure 3:**
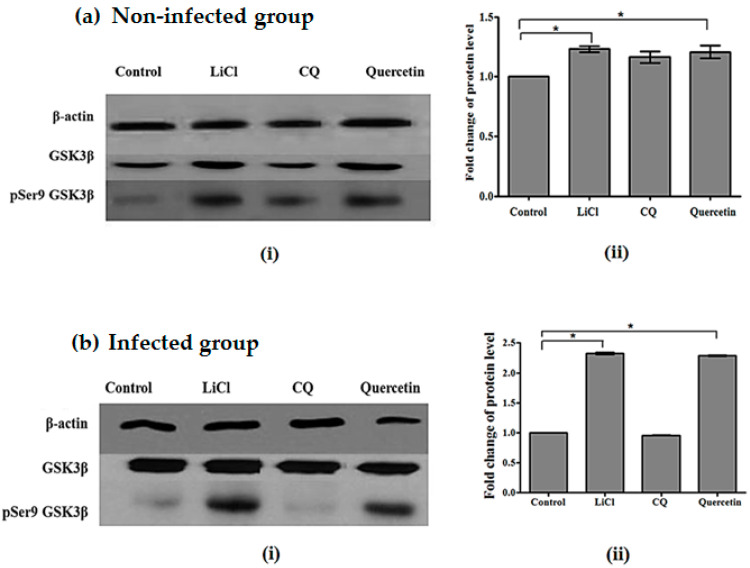
(**a**) (i) Intensity of immunoreactive bands for pSer9 GSK3β and GSK3β (ii) Fold change of phosphorylated protein levels in the brain of non-infected mice; (**b**) (i) Intensity of immunoreactive band for pSer9 GSK3β and GSK3β (ii) Fold change of phosphorylated protein levels in mice infected with *P. berghei* ANKA treated with LiCl, CQ, or quercetin for four days. Total GSK3β protein expression levels were normalised to β-actin, and pGSK3β was normalised to total GSK3β protein level. Densitometric measurements are illustrated as mean (fold) ± SD of corresponding measurements in non-treated controls. Significant differences between control and test groups were measured at *p* < 0.05 (*).

Western-blot analysis for non-infected animals showed the presence of GSK3β and pSer9 GSK3β in non-infected brain samples, as seen from the immunoreactive-GSK3 and -pSer9 GSK3 protein bands. The intensities of the immunoreactive bands of GSK3β were similar, indicating that the total GSK3β protein levels were the same in the treatment group and control group. The intensities of immunoreactive bands for pSer9 GSK3β were higher for quercetin- and LiCl-treated groups. Animals treated with quercetin and LiCl each showed significant increases in pSer9 GSK3β with an increment of 1.2-fold for both treatments compared to the control group. Meanwhile, immunoreactive-pSer9 GSK3β protein bands for CQ have lower intensities as compared to quercetin- and LiCl-treated groups (Figure 3).

As for *P. berghei* ANKA-infected groups, western analysis of brain samples showed that GSK3β and pSer9 GSK3β were present as immunoreactive-GSK3β and -pSer9 GSK3β protein bands. Uniform intensities in immunoreactive-GSK3β protein bands indicate the same level of total GSK3β protein in both treated and control groups. Figure 3 shows a significant fold increase of pSer9 GSK3β protein in the brain sample of infected mice. *P. berghei* ANKA-infected mice treated with quercetin and LiCl each showed a significant increase in the fold change of pSer9 GSK3β with a 2.3-fold increment for both treatments compared to non-treated controls.

### 2.6. Quercetin Modulated Pro- and Anti-Inflammatory Cytokine Levels in P. berghei NK65-Infected Animals

**Figure 4:**
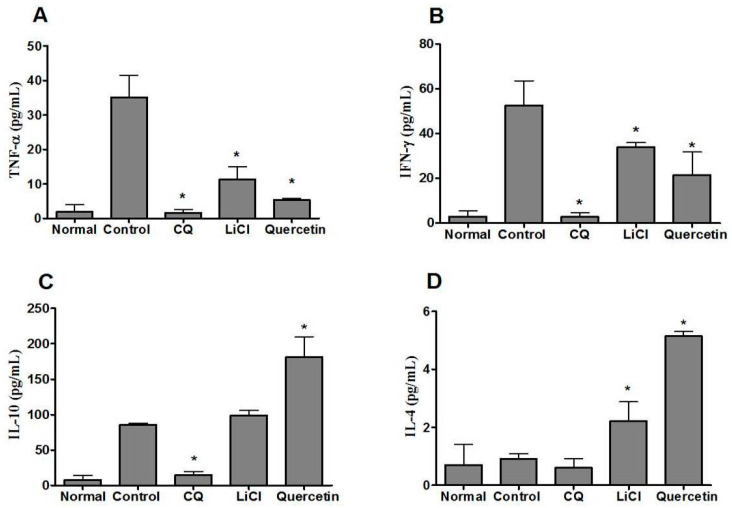
At day 4, post-infection of *P. berghei* NK65, pro-inflammatory cytokine (**A**) TNF-α, (**B**) IFN-γ and anti-inflammatory cytokines (**C**) IL-10 and (**D**) IL-4 levels in serum of mice administered with CQ, LiCl, or quercetin. Data represent mean ± SD of normal (non-infected), control (infected), and CQ/LiCl/quercetin-treated infected mice (*n* = 7). Significant differences between control (infected) and treatment groups were determined at *p* < 0.05 (*).

Inflammatory cytokine measurements indicated that there were increases in both pro- and anti-inflammatory cytokine levels on day four after *P. berghei* NK65 infection. Upon parasite infection, levels of pro- and anti-inflammatory cytokines TNF-α, IFN-γ, IL-10, and IL-4 were increased by 7.0, 10.0, 14.0, and 1.5 times, respectively, in the systemic blood circulation of infected mice (Figure 4). On day four post-infection, the levels of pro-inflammatory cytokines TNF-α and IFN-γ were decreased to 6.6 and 2.5 times that observed in controls respectively while the levels of anti-inflammatory cytokine IL-10 and IL-4 were increased (by 2.1 and 5.7 times) upon quercetin treatment (Figure 4). As for mice-treated with LiCl, levels of TNF-α and IFN-γ decreased to 3.1 and 1.6 times that in controls, respectively. Level of IL-10 and IL-4 increased to 1.2 and 2.4 times that in controls, respectively. For CQ treatment, levels of pro-inflammatory cytokines TNF-α, IFN-γ, IL-10 and IL-4 were also decreased significantly after infection (to 12.0, 25.0, 9.0, and 1.5 times that in controls, respectively). CQ has anti-parasitic and anti-inflammatory properties, which can directly inhibit the growth of parasite at the early stage of infection and exert a direct effect on the host immune system [42,43].

## 3. Discussion

Findings from our in vitro studies showed that quercetin exhibited moderate anti-plasmodial activity with a high selectivity index. This observation that quercetin exhibits in vitro anti-plasmodial activity corroborates previous reports by other investigators [28]. Another study [44] showed that aminoalkylated-quercetin analogues exhibited strong anti-malarial activities with IC_50_ values within the nanomolar to low micromolar range against *P. falciparum* W2, D6, and C235. Dysregulation of GSK3β during infection can result in overwhelming production of pro-inflammatory cytokines [32]. Thus, inhibitors of GSK3 not only can potentially serve as anti-malarial agents but also as cytokine modulators [45]. During malarial infection, excessive pro-inflammatory response contributes to the pathophysiology of the disease [3]. A feasible therapeutic approach in host-directed therapy is to mitigate the overwhelming cytokine response. GSK3 inhibitors as therapeutics potentially afford dual benefits [17], thus suitable to be explored in the treatment of malaria, i.e., inhibiting parasite growth and concomitantly modulating cytokine balance.

Findings from the present in vivo studies demonstrated that quercetin has anti-malarial activities as well as a cytokine-modulating effect. Our study revealed that following quercetin treatment, there is significant reduction in the levels of pro-inflammatory cytokines; TNF-α and IFN-γ and increased levels of anti-inflammatory cytokines; and IL-10 and IL-4 evidence of quercetin-modulated pro- and anti-inflammatory cytokine levels in *P. berghei* NK65-infected animals. Although the anti-malarial effect of quercetin has been documented previously by other investigators, the present study represents the first report of the dual anti-malarial and cytokine-modulating activities of quercetin. The anti-malarial effect of quercetin demonstrated here is only moderately potent compared to CQ; nevertheless, it is envisaged that the dual anti-malarial and cytokine-modulating effects of quercetin render it a potential adjunctive therapeutic. Based on its anti-inflammatory and anti-plasmodial activity for malaria, quercetin, when combined with current anti-malarials, may be useful as a new and more effective preventive and therapeutic agent against malaria, albeit subject to a clinical trial. Quercetin acted not only in suppressing the intra-erythrocytic stage of *P. berghei* NK65 but also in improving animal survivability. It is tempting to speculate that the increase in mean survival time observed in the present study is at least in part attributed to quercetin mitigating the cytokine storm.

In this study, based on the western analysis, we have also demonstrated that treatment with quercetin resulted in increased pGSK3β (Ser9) and thus inhibition of GSK3β activity in the liver of quercetin-treated NK65-infected animals. Since GSK3β is involved in the innate and adaptive immune response through the regulation of cytokine production [32], higher GSK3β in the liver of untreated NK65-infected mice may be associated with increased pro-inflammatory cytokine production during inflammation upon parasite infection. Quercetin treatment in *P. berghei* NK65-infected animals resulted in significant reduction in the levels of pro-inflammatory cytokines; TNF-α and IFN-γ and increased levels of anti-inflammatory cytokines; and IL-10 and IL-4. This balancing in the production of cytokines may be a consequence of the inhibitory effect of quercetin on GSK3β, a kinase that plays a pivotal role in mediating inflammation [46]. These findings suggest that quercetin modulates the level of inflammatory cytokines through phosphorylation and consequent inhibition of GSK3β in the liver of NK65-infected animals. Besides, GSK3β inhibition is known to drive the maturation and function of natural-killer (NK) cells [47] and thus can contribute to parasite clearance. As demonstrated in this study, quercetin treatment displayed chemo-suppressive effects in *P. berghei*-infected animals. This study is not the first report on a plant-derived compound that can inhibit host GSK3β in malaria-infected animals. We have previously reported that curcumin, an active anti-inflammatory agent, inhibited GSK3β in the liver of NK65-infected mice. Also, in our laboratory, we have previously shown that the anti-malarial effects of kaempferol (also a flavone and structurally very similar to quercetin) involved cytokine modulation mediated through inhibition of host GSK3β [48]. Subsequently, Somsak et al. [49] demonstrated that combination treatment using CQ and kaempferol presented significant anti-malarial effects. Recently, Ounjaijean et al. [50] reported that a combination of kaempferol and artemisinin exerted potent anti-malarial activity with a synergistic effect against PbANKA-infected mice and suggested this effect could be attributed to the anti-oxidant and anti-cancer activities of kaempferol. On the other hand, it is tempting to speculate that the anti-inflammatory and GSK3β-inhibitory properties of kaempferol possibly also contribute to the above reported anti-malarial effects. Thus, flavonoids exhibiting GSK3β-inhibitory properties can be exploited for the development of therapies for malaria.

Our western-blot analysis also revealed that pSer9 GSK3β protein level is higher in the brain of *P. berghei* ANKA-infected mice treated with quercetin as compared to the control group. Our results showed that quercetin treatment was able to inhibit the GSK3β in the brain of ANKA-infected mice. Quercetin has also been reported to have the capability to infiltrate the blood–brain barrier (BBB) in mice, which allows it to be an effective neuroprotective agent [51,52]. The capability of this flavonoid penetrating the BBB and inhibiting GSK3β in the brain of ANKA-infected mice may be one of the reasons that this compound prolonged animal survivability as demonstrated in our present study.

High levels of pGSK3β (pSer9 GSK3β) in LiCl-treated infected mice were also observed in NK65-infected animals, indicating that GSK3β is inhibited. LiCl, a GSK3 inhibitor used as a reference inhibitor in this experiment, inhibited GSK3β and reduced the production of pro-inflammatory cytokines during infection. LiCl is reported to directly inhibit GSK3β through Akt activation via the PI3K signalling pathway [53,54]. LiCl has been shown to cause direct inhibition of GSK3 through GSK3 *N*-terminal phosphorylation as observed from rapid Ser9 phosphorylation in 293T, Neuro2A, and NIH3T3 cells [55]. The fold change of pSer9 GSK3β in quercetin-treated mice is similar to that of LiCl-treated mice, suggesting that in vivo, quercetin has a GSK3β-inhibitory effect that is comparable to LiCl. Our previous study showed that LiCl suppressed the development of parasitaemia in *P. berghei* NK65-infected animals [56]. A subsequent study by Dai et al. [57] showed that LiCl was able to modulate the levels of pro- and anti-inflammatory cytokines in the brain of ANKA-infected mice through GSK3β inhibition.

Findings from our study revealed that quercetin and chloroquine treatments each decreased the levels of pro-inflammatory cytokines TNF-α and IFN-γ significantly. As for the anti-inflammatory cytokines, IL-4 and IL-10, only quercetin (and not chloroquine) treatment was observed to increase the levels, therefore suggesting that quercetin and chloroquine may be working via different mechanisms in affecting the (anti-inflammatory) cytokine levels. The cytokine-modulatory effect of quercetin observed here renders it different from conventional anti-malarial agents, which in most cases, only exhibit anti-parasitic activity. Recently however, chloroquine was shown to exhibit not only anti-parasitic activity but also anti-inflammatory properties and thus may directly affect the host immune system [42]. The mechanism of host-mediated response due to chloroquine is however still not fully understood [58]. Immune mechanisms associated with chloroquine treatment can be different between human and animal infections. For example, in a controlled human–malaria study, IFNs were shown to regulate immune response by promoting IL-10 to suppress inflammatory cytokines; additionally, parasite-specific IL-10 production was observed in *P. falciparum* patients in the field following chemoprophylaxis with chloroquine or artemisinin [59].

Furthermore, LiCl treatment restored long-term neurocognitive function in experimental cerebral malaria suggesting that the anti-malarial effect is attributed to the dysregulation of GSK3β [57]. The most serious and increasingly lethal neurological complication of infection with *P. falciparum* is cerebral malaria, responsible for over two million deaths annually. In terms of minimizing neurological and cognitive impairments, the current treatment is insufficient [60]. The pathophysiology of cerebral malaria is complicated. Nevertheless, GSK3 may be one of the plausible targets to be exploited in adjunctive therapy to overcome neurological deficits. Recently, Worthen et al. [61] demonstrated that anti-inflammatory IL-10 administration rescues depression-associated learning and memory deficits in mice. As mentioned earlier, Dai et al. [57] reported that LiCl treatment restored long-term neurocognitive function in experimental cerebral malaria. Thus, increased IL-10 production in the brain as a consequence of GSK3β inhibition could have helped restore neurocognitive impairment in the above findings in experimental cerebral malaria.

The cytokine-modulatory effect of quercetin reported renders it different from the conventional anti-malarial agents, which in most cases, only exhibit anti-parasitic activity. There is an urgent need for new therapeutic approaches for malaria to mitigate the cytokine storm during the early infection phase instead of only focusing on eliminating the parasite. Inhibitors of GSK3β has the potential to simultaneously target both parasite growth and overwhelming immune response against *P. falciparum*.

## 4. Materials and Methods

### 4.1. Parasites

The human malarial parasite, *P. falciparum* 3D7 (chloroquine-sensitive) (MRA-102) and the rodent malarial parasites, *P. berghei* NK65 (MRA-268) and *P. berghei* ANKA (MRA-671), were originally obtained from BEI Resources, NIAID, NIH (*P. falciparum*, Strain 3D7, MRA-102, contributed by Daniel J. Carucci; *P. berghei*, Strain NK65, MRA-268, contributed by Victor Nussenzweig; *P. berghei*, Strain ANKA, MRA-671, contributed by M. F. Wiser).

### 4.2. In Vitro Anti-Plasmodial Assessment

*P. falciparum* 3D7 was cultured in Roswell Park Memorial Institute-1640 (RPMI-1640) culture medium in the asynchronised phase at 2% haematocrit and 2% parasitaemia in 100 µL of quercetin at concentrations ranging from 0.0001 to 100 µM. Parasitised red blood cells without treatment served as the positive control. As a negative control, un-parasitised O+ red blood cells with no treatment were used in the assay. The concentration of the test compound used in the study ranged from 0.0001 to 1000 µg/mL. The anti-malarial reference drug CQ used ranged from 0.0001 to 10 µM. The assay was based on the lactate dehydrogenase enzymatic reaction (pLDH) [62,63]. After 48 h incubation of *P. falciparum* cultures with test compounds, Malstat and NBT/PES reagents were added to allow colour changes. Absorbance readings were taken at 650 nm using a spectrophotometer (Fluorostar OPTIMA, Ortenberg, Germany). Data were analysed for IC_50_ values (inhibition concentration at 50% parasite growth) through non-linear regression.

### 4.3. In Vitro Cytotoxicity Assessment

Chang liver cells were purchased from the American Type Culture Collection (ATCC Manassas, VA), USA. Cytotoxicity of quercetin was measured using the 3-(4, 5-dimethylthiazol-2-yl)-2, 5-diphenyltetrazolium bromide (MTT) assay [64,65]. Chang liver cells were seeded at 2 × 10^4^ in Dulbecco’s Modified Eagle Medium (DMEM). The concentration of the test compound used in the assay ranged from 0.0001 to 1000 mg/mL. Cell suspension without test compound was set as positive control. The culture was incubated with or without test compound for 48 h (37 °C, 5% CO_2_). Then, MTT–PBS reagent was aliquoted into each well. The plates were incubated for 3 h at 37°C. The mixture was removed and replaced with dimethyl sulphoxide (DMSO) to dissolve the MTT formazan product. The mixture was mixed for 15 min, and absorbance measured at 540 nm (Fluorostar OPTIMA). IC_50_ values were calculated through a non-linear regression curve. Selectivity index (SI) values were calculated based on the following formula: SI = (IC_50_ MTT assay)/(IC_50_ pLDH assay).

### 4.4. Experimental Animals

Male ICR mice weighing 20–25 g provided by the Animal House Complex, Universiti Kebangsaan Malaysia (UKM) were used. Permission and approval for animal studies were obtained from the Universiti Kebangsaan Malaysia Animal Ethics Committee (UKMAEC) (reference number: FST/2015/HASIDAH/11-FEB./640-FEB.-2015-DEC.-2016).

### 4.5. Survivability Test

In order to ensure that the dosage of quercetin (Sigma, St. Louis, MO, USA) used in the four-day suppressive test did not impact survival and toxicity of the experimental animals, survivability and behavioural changes were monitored. A total of 49 healthy male ICR mice (*n* = 7) were injected (intraperitoneal (i.p.)) with 2.5, 5, 10, 15, 25, and 50 mg/kg body weight (BW) quercetin daily for four consecutive days. Quercetin was freshly prepared by first dissolving 50 mg with 1 mL of DMSO (Sigma, St. Louis, MO, USA) then diluting it to the desired concentrations using normal saline (0.85% (*w/v*) NaCl solution). The final percentage of DMSO in the solution did not exceed 10% to ensure no toxic and harmful effect to the animals. Each mouse was administered with quercetin in 0.2 mL injection volume. NaCl solution (0.85% (*w/v*)) was administered to the control group of animals. Gross behavioural changes (e.g., diarrhoea, excess urination, lethargy, or reduction in locomotor activities) and survivability of all groups were monitored within 30 days.

### 4.6. Four-Day Suppressive Test

The anti-malarial activity of quercetin was assessed in male ICR mice according to the four-day suppressive test [66,67]. Mice were randomly divided into eight groups (*n* = 7) for *P. berghei* NK65 infection. Each mouse was inoculated i.p. with 0.2 mL of 2×10^7^ of pRBC (parasitised red blood cells) containing *P. berghei* NK65. The animals infected with NK65 strain were administered i.p. with 2.5, 5, 10, 25, and 50 mg/kg BW/day quercetin. In a separate experiment, animals were randomly divided into six groups (*n* = 7) for *P. berghei* ANKA infection. Each mouse was inoculated i.p. with 0.2 mL of 2×10^7^ of pRBC containing *P. berghei* ANKA. The animals infected with ANKA strain were then administered i.p. with 15, 25, and 50 mg/kg BW/day quercetin. For both infections, CQ at 10 mg/kg BW/day was used as anti-malarial reference drug (positive control), LiCl at 100 mg/kg BW/day as GSK3 inhibitor reference drug, and an injection of 0.2 mL of 0.85% (*w/v*) NaCl to the infected animals as non-treated controls (negative control). Mice were administered with test compounds for four consecutive days, starting at one hour after parasite inoculation on day 0 (D0). Thin blood smears were prepared at day 4 (D4) from tail blood of infected mice. The percentage of chemo-suppression (PC) (%) was then determined by using the following formula:

PC = 100 × [(A − B)]/A (1)

where A represents the percentage of parasitaemia (%) of non-treated negative control and B represents the percentage of parasitaemia (%) in test groups.

### 4.7. Western-Blotting Analysis

On day 4 (D4) post-infection, *P. berghei* NK65-infected mice were sacrificed for liver collection, while *P. berghei* ANKA-infected animals were sacrificed for the brain. Each organ was rinsed with ice-cold pH 7.2 PBS solution and dried on 3 mm Whatman paper. The organs were homogenised on ice with cold extraction buffer (RIPA buffer (150 mM NaCl, 1% Triton X-100 (*v*/*v*), 0.5% sodium deoxycholate (*w/v*), 0.1% SDS (*w/v*), 50 mM Tris-HCl pH 8.0, and 1 mM EGTA)) and phosphatase inhibitor (15 mM NaF, 1 mM Na_3_VO_4_, and 5 mM EDTA). Samples were then centrifuged at 12,000× *g* for 30 min at 4 °C (Eppendorf, Model 5810R, Hamburg, Germany). Total protein in the organ extracts were determined using the Bradford method [68].

Protein separation was done using sodium dodecyl sulfate polyacrylamide gel electrophoresis (SDS-PAGE) [69]. Equal amounts of liver protein samples (40 µg) were loaded into each well of SDS-PAGE gels (30% acrylamide). The separated proteins on the gel were then transferred onto nitrocellulose membrane Hybond ECL (GE Healthcare, Amersham, UK). The membrane was incubated with primary monoclonal antibodies, anti-GSK3β, anti-phosphoSer9-GSK3β or β-actin (Cell Signaling Technology, Beverly, MA, USA) followed by a 2-h incubation with corresponding secondary antibody, anti-rabbit IgG HRP-linked antibody (Cell Signaling Technology, Beverly, MA, USA) at room temperature. Detection of immunoreactive proteins was carried out using enhanced chemiluminescence reagent (ECL) (Thermo Scientific, Waltham, MA, USA) in the dark. Band-area-intensity quantification was performed using a densitometer (Vilber Lourmat, Marne-la-Vallée, France).

### 4.8. Serum Cytokine Analysis

For groups of animals infected with *P. berghei* NK65 and controls, serum cytokine levels were assessed. At day four post-infection, blood was collected using cardiac puncture from euthanised animals (*n* = 7) and immediately centrifuged to obtain serum. Levels of pro-inflammatory cytokines (TNF-α and IFN-γ) and anti-inflammatory cytokines (IL-10 and IL-4) were measured in sera of treated and control animals using an enzyme-linked immunosorbent assay (ELISA) (QIAGEN, Hilden, Germany).

### 4.9. Statistical Analysis

Variance analysis and log-rank test (for Kaplan–Meier survival analysis) were conducted to assess the statistical significance of the data between groups. Data obtained are expressed as mean ± standard deviation (SD). A P value of <0.05 between groups was considered to be significant.

## 5. Conclusions

Findings from our study demonstrate that quercetin can elicit anti-malarial and GSK3β-mediated cytokine-modulating effects. The dual anti-plasmodial and GSK3β-mediated cytokine-modulating activities of quercetin are requisite of its potential as a plant-derived therapeutic in malaria. Quercetin and its derivatives are plausible compounds to be tested in clinical trials. Our findings also reiterate that GSK3β is a plausible anti-malarial target for adjunctive therapy.

## Figures and Tables

**Table 1:** Quercetin-suppressive activity in *P. berghei* NK65 and ANKA-infected mice.

*P. berghei* strain	*P. berghei* NK65			*P. berghei* ANKA		
Compound/Drugs	Dosage (mg/kg BW)	Parasitaemia Suppression on Day 4 (%)	Median Survival Time (Days)	Dosage (mg/kg BW)	Parasitaemia Suppression on Day 4 (%)	Median Survival Time (Days)
Quercetin	2.5	46.9 ± 3.5 ^a^	9 ^b^	15	36.1 ± 5.7 ^a^	10 ^a^
	5	51.1 ± 5.7 ^a^	13	25	24.9 ± 3.8 ^a, b^	9 ^b^
	10	53.5 ± 4.3 ^a^	14	50	24.3 ± 1.8 ^a, b^	9 ^b^
	25	60.7 ± 2.4 ^a^	17 ^a^			
	50	49.3 ± 3.6 ^a^	17 ^a^			
CQ (Anti-malarial reference drug)	10	96.0 ± 1.1 ^a^	>30 ^a^	10	94.3 ± 1.8 ^a^	>30 ^a^
LiCl (GSK3 inhibitor reference)	100	69.4 ± 3.2	15 ^a^	100	85.2 ± 0.9 ^a^	10 ^a^
0.85% (*w*/*v*) NaCl (Negative control)	0.2 mL	-	13	0.2 mL	-	7

Data represent mean ± SD for parasitaemia suppression and median survival time (*n* = 7). ^a^ represents values significantly different from negative control (*p* < 0.05), ^b^ represents value which significantly different from CQ (*p* < 0.05).

## Data Availability

The data presented in this study are available in this article.
